# Engineering principles in plant metabolism: integrating control, mechanics, and transport under dynamic environments

**DOI:** 10.1093/plphys/kiag535

**Published:** 2026-08-03

**Authors:** Tim Nies, Josha Ebeling, Anna Matuszyńska

**Affiliations:** Computational Life Science, Department of Biology, RWTH Aachen University, Aachen 52074, Germany; Computational Life Science, Department of Biology, RWTH Aachen University, Aachen 52074, Germany; Computational Life Science, Department of Biology, RWTH Aachen University, Aachen 52074, Germany; Center for Computational Life Sciences, RWTH Aachen University, Aachen 52074, Germany

## Abstract

Plant responses to dynamic environmental conditions remain challenging to describe within a unified mechanistic framework. This is largely because metabolism operates under simultaneous and interacting regulatory, mechanical, and transport constraints. Here, we synthesize three engineering perspectives as complementary frameworks to quantify these constraints on plant metabolism: (i) **control engineering**, for dynamic regulation of the biochemical pathways; (ii) **structural and mechanical engineering**, to quantify load-bearing constraints and geometry-dependent scaling relationships in plant tissues; and (iii) **fluid dynamics**, which describes xylem and phloem transport as flow through networks under variable demand. For each domain, we present plant-specific case studies demonstrating how engineering analysis can reveal constraints and dynamic relationships that are difficult to extract from descriptive approaches alone, and we explicitly distinguish quantitatively validated examples from instructive structural analogies. Photosynthetic carbon fixation under fluctuating conditions serves as a recurring example, as it simultaneously involves feedback regulation, mechanical stomata control, and hydraulic water supply. To overcome the limitations of purely mechanistic models as system complexity increases, we discuss emerging hybrid modeling strategies that combine mechanistic understanding with machine learning. These approaches facilitate parameterization and cross-scale integration, providing a foundation for future predictive digital twins of plant metabolism. Finally, we present a strengths, weaknesses, opportunities, and threats analysis of key limitations arising from nonlinearity, heterogeneity, and measurement constraints, and conclude by outlining open experimental and modeling questions at the interface of plant metabolism and engineering. We aim to help plant biologists translate their biological questions into predictive, multi-scale models and, ultimately, digital representations of plant metabolism.

## Introduction

Plants grow, photosynthesize, transport resources, and respond to environmental stimuli through tightly regulated metabolic processes. Over decades, plant scientists have built rich descriptive knowledge of metabolism, from photosynthetic mechanisms to stem biomechanics. However, moving from qualitative descriptions to quantitative predictions under dynamic real-world conditions remains challenging (Morris and ten Tusscher 2021). Natural environments impose continuous, time-varying stresses, fluctuating light, mechanical loads, and changes in water availability that often interact nonlinearly and in a state-dependent manner, amplifying system responses (Rillig et al. 2021; Zandalinas et al. 2021; Zandalinas and Mittler 2022). The central challenge is therefore not describing individual processes, but predicting their coupled dynamics. This requires frameworks that explicitly account for temporal variability, feedback regulation, and physical constraints across scales.

Engineering disciplines provide such frameworks. Developed to design and analyze systems under constraints and perturbations, they offer mathematical formalism for describing dynamic behavior and performance (Vincenti 1997; Franssen et al. 2024), and excel at iterative design-test-refine cycles (Wynn and Eckert 2017). Applied to plant systems, they enable metabolism to be formulated not only as a reaction network but as a dynamical system constrained by regulation, structure, and transport. Questions such as *how substrate concentrations are stabilized*, *how structural design constrains metabolic activity*, or *how resource distribution adapts across tissues* can thus be posed in quantitative, system-level terms. Constraint-based approaches, including flux balance analysis, formulate metabolism as a constrained optimization problem and have proven powerful for mapping metabolic capabilities and steady-state flux distributions (de Oliveira Dal’Molin et al. 2010; Sweetlove and Ratcliffe 2011). They can be extended toward environmentally perturbed plant metabolism, bridging genome-scale metabolic modeling and stress-response analysis (Töpfer 2021). However, they generally do not resolve regulatory dynamics, transient feedbacks, or spatial-mechanical processes shaping responses over time. These aspects require complementary dynamic modeling frameworks that represent time-dependent regulation, mechanical limits, and spatial transport (Wendering and Nikoloski 2023). Stomatal regulation under variable light illustrates this gap: carbon gain depends not only on metabolic capacity, but also on how rapidly and accurately signaling, ion transport, and hydraulic processes adjust to changing conditions (Buckley 2017; Yang et al. 2020; Joshi et al. 2022; Bahadar et al. 2025).

Links between structure and function have long been recognized in plant science. Early work on plant movement (Darwin and Darwin 1880) and canopy architecture (Monsi et al. 1973) established that geometry and mechanics shape physiological performance and metabolism. These relationships were later formalized using structural engineering concepts, modeling petioles as cantilevered beams or leaf surfaces as deformable structures (Niklas 1999). Advances in mechanical metrology have since enabled direct, quantitative measurements of material properties across scales, from cell walls to small organs (Moulia 2013; Shah et al. 2017), providing parameters required to integrate mechanical constraints into dynamic descriptions of plant function.

In this review, we synthesize three engineering domains that together characterize the principal physical constraints on plant metabolism under dynamic conditions. *Control engineering* addresses how plants regulate metabolic states through feedback. *Structural and mechanical engineering* addresses how load-bearing constraints, material properties, and geometry determine feasible metabolic configurations. *Fluid dynamics* addresses how transport capacity, flow resistance, and pressure gradients constrain supply and product distribution across distances. Three complementary glossaries (Text Boxes 1–3) define key engineering concepts.

**Figure 1:**
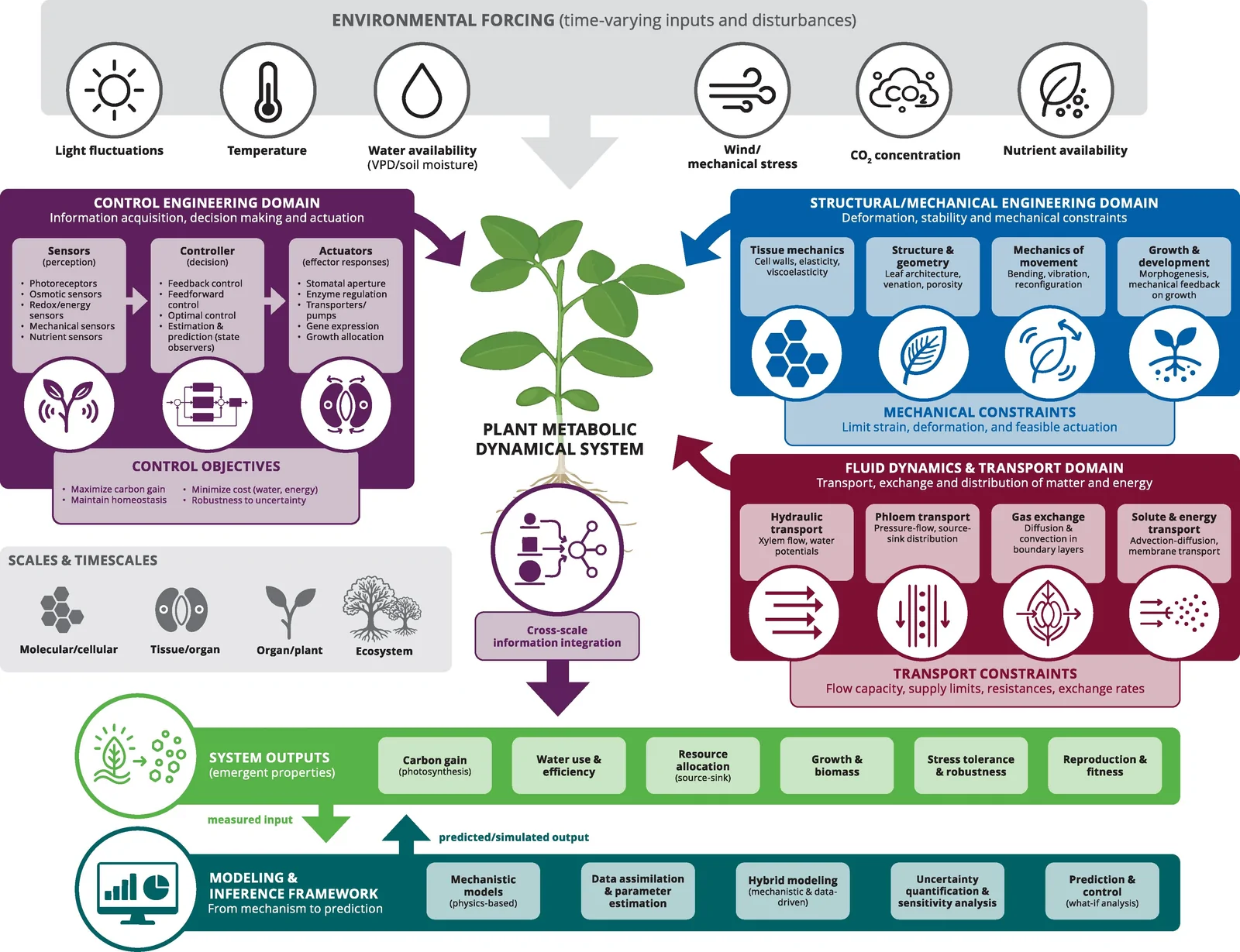
Engineering frameworks for the quantitative analysis of plant metabolism across scales. Three engineering domains—control engineering (left box, purple), structural and mechanical engineering (right upper box, blue), and fluid dynamics (right, middle box, red)—address plant metabolism from carbon acquisition to cellular regulation, under time-varying environmental inputs including light, temperature, water availability, wind, and carbon dioxide concentrations (top box, gray). As a dynamic metabolic system, plants respond to environmental changes by activating various metabolic processes across time and spatial scales (middle gray box). Control engineering describes information acquisition, feedback regulation, and actuation underlying metabolic homeostasis and carbon gain. Structural/Mechanical Engineering quantifies how load-bearing constraints and material properties determine plant movement, stability, and light interception. Fluid dynamics characterizes how transport capacity, flow resistance, and pressure gradients constrain metabolite distribution through xylem, phloem, and cytoplasm. Integrating the effects of all abiotic factors results in characteristic changes in system outputs, such as carbon gain, resource allocation, and fitness (second from the bottom box, light green). By combining these outputs with a range of modeling and inference frameworks, researchers can develop computational representations of plants. These representations integrate engineering principles, mechanistic understanding, and data analysis, enabling advanced computational analyses using digital twins that inform the prediction and control of, for example, crop yield (bottom box, dark green).

These three domains are not independent: they are coupled at the spatial and temporal scale of plant metabolism (Fig. 1). Hence, after we discuss control, mechanics, and transport separately, we integrate them using photosynthetic carbon gain under fluctuating environments as a synthesis example. We end by considering hybrid modeling approaches, combining mechanistic structure with machine learning (ML), as main enablers of cross-domain integration and predictive *digital biotwins* generation, and discuss the strengths and limitations of applying engineering frameworks in plant science. The review is selective rather than systematic. We prioritize examples where engineering concepts have been explicitly applied to plant systems, distinguishing validated models from structural analogies (Table 1).

**Table 1:** Biological questions in plant metabolism addressed using engineering concepts.

Biological question	Engineering framework	Key assumption	Engineering contribution	Empirical validation status	Reference
How is intracellular nitrate homeostasis achieved?	Controllers (CE)	Single-E controller	Derives differential equation models of inflow–outflow control motifs that predict steady-state nitrate concentration under perturbation and quantifies how controller accuracy depends on parameters.	Computational simulations qualitatively reproduce steady-state nitrate concentrations and fluxes in barley root cells	Huang et al. (2012)
How is intracellular iron homeostasis maintained?	Controllers (CE)	Single-E controller	Derives differential equation models of inflow–outflow control motifs predicting stable cytosolic iron concentration and determines how iron concentration is maintained by a hierarchy of different set points based on storage, outflow, and inflow.	Computational simulations qualitatively reproduce mRNA and protein-levels in *Arabidopsis thaliana*	Hussey et al. (2013)
How are hormone concentrations stabilized in signaling networks?	Controllers (CE)	Double-E controller	Proposes an enzyme-catalyzed double-E controller mechanism that explains brassinosteroid homeostasis via removal of phosphorylated receptors.	Conceptual analogy	He et al. (2026)
How is redox balance maintained in the photosynthetic electron transport chain?	Controllers (CE)	Time scale separation, linearized system, zeroth-order protein degradation	Maps proportional-integral control equations onto concrete molecular mechanisms, identifying transcriptional regulation of Lhcb genes and transfer of LHCII redistribution as implementation of regulatory processes that provide a mechanistic explanation for PQ redox stabilization.	Conceptual analogy	HW et al. (2002)
How do photosynthetic and genetic systems respond to fluctuating environmental inputs across timescales?	FQ-domain, TF / Bode plots (CE)	Linearized systems/operation in the linear domain or system can be described by a sum of sinusoids	Quantifies frequency-dependent gain and phase-shift of photosynthetic responses as a function of light input frequency and amplitude using frequency-domain analysis. Linearize and transform dynamic models of genetic processes to fully characterize input–output relationships.	Photosynthesis: Validated in plants using fluorescence data Genetic: Conceptual analogy	Kacser and Burns (1973), Kacser et al. (1995), Jensen et al. (2012), Katifori (2018), Jones et al. (2022), Kaňa et al. (2023)
What regulatory mechanisms determine the dynamic response of photosynthesis to changing inputs?	System identification (CE)	Processes can be decomposed in nonlinear and linear parts or can be locally assumed to be linear	Derives input–output models from photosynthetic data, quantifying response characteristics of underlying regulatory processes to fluctuating light.	Computational simulations qualitatively reproduce chlorophyll fluorescence in plants and *in silico* datasets	Khodarahmi and Maihami (2023), Kibrete et al. (2023)
What mechanical properties determine leaf movement, and how does leaf movement relate to photosynthesis?	Beam theory (SE)	Linear homogeneous beam	Computes leaf bending stiffness, curvature, and deflection under external loads using beam theory and elastic sheet models, linking structural constraints on leaf orientation to the function of biological processes, e.g. photosynthetic light capture.	Validated in plants by comparing theory with experimental measurements of, for instance, leaf position and flexural rigidity.	Knoblauch and Oparka (2012), Briat et al. (2018)
What structural properties determine the response to wind, and how does the trunk swaying relate to metabolic processes?	Beam theory (SE)	Linear homogeneous beam	Quantifies stem deformation and describes adaptation of stiffness or stem taper to mechanical forces for increased plant stability.	Validated in plants by comparing theory with experimental measurements of stem form	Lauderbaugh et al. (2021), Lauderbaugh and Holder (2022)
How do roots anchor plants, and what mechanical forces determine root protrusion?	Beam theory (SE)	Linear homogeneous beam	Derives models to quantify the deformation of roots in soil, root stiffness, and anchorage of trees under varying environmental loads and interprets experimental measurements on root growth and soil interaction to assess mechanical constraints on plant stability.	Validated in plants by comparing theory with experimental data of root form, load-displacement and shear test data	Launder and Spalding (1974), Laanemets et al. (2013), Lavarenne et al. (2018)
How do the mechanical properties of the cytoskeleton relate to the efficiency of cargo transport?	Beam theory (SE) and material design (ME)	Linear homogeneous beam	Quantifies the mechanical properties of cytoskeletal filaments, treating them as stiff beam-like structures, and determines mechanical constraints on intracellular transport imposed by filament bending, curvature, and geometry.	Validated outside higher plants by comparing theory with experimental data of young moduli, polymer form, and flexural rigidity	Milo et al. (2002), Nedbal and Březina (2002), Nedbal et al. (2005), Monshausen and Haswell (2013), Nasrin et al. (2020), Nasrin et al. (2021)
How can mechanical forces be translated into intracellular signals, and to what extent do mechanical properties determine the signal translation?	Beam theory (SE) and material design (ME)	Linear homogeneous beam	Provides a mechanical framework for understanding force perception through cytoskeletal properties and other mechanosensitive structures and its impact, e.g. on developmental changes in meristematic tissue.	Validated in plants by investigating, for instance, the effects of mechanoperception in *A. thaliana*	Niklas (1999), Nardini et al. (2011), Nedbal and Lazár (2021)
How do material properties of biomembranes contribute to cell form and intracellular compartmentalization?	Material design (ME)	Membrane is a thin, homogeneous sheet. Elastic energy depends on curvature and area	Maps the mechanical properties of biomembranes, such as bending elasticity and tension, to cell shape and compartment integrity	Validated outside higher plants measuring membrane tension and comparing theory to the form of cells	Oishi and Klavins (2011), Pritchard and Birch (2011)
How effective is cytoplasmic streaming for metabolite transport?	CFD (FD), Hydrodynamics	Low Reynolds number regime and incompressible fluid	Computes flow patterns of metabolites in cells and derives how cytoplasmic streaming affects metabolism, enabling predictions of substrate distributions in algal cells	Computational simulations reproduce fluid flow in cells	Thorsen et al. (2013), Thorsen et al. (2019), Montefusco et al. (2024)
What physical constraints govern long-distance transport in plants?	CFD (FD), Hydrodynamics	Pressure-driven (positive and negative) flow	Quantifies flow constraints in xylem and phloem, revealing physical limits on transport efficiency, refilling, and growth.	Computational simulations qualitatively reproduce water and solute transport in plants (where possible)	Shoval and Alon (2010), Shelley (2024), Shteinberg et al. (2024), Smoleňová et al. (2025), Siska et al. (2026)

Abbreviation: CE—control engineering, FQ—frequency, TF—transfer function, SE—structural engineering, ME—mechanical engineering, CFD—computational fluid dynamics, FD—fluid dynamics.

## Control engineering for metabolic regulation

Control engineering studies how systems maintain stable outputs in response to defined inputs (Nise 2019), designing feedback loops that regulate behavior while ensuring robustness against disturbances. Biological systems rely on analogous control mechanisms to regulate metabolic, genetic, or physiological processes, a parallel particularly evident in molecular control systems. This similarity offers an opportunity for knowledge transfer between plant science and engineering, especially in metabolism, where control operates across hierarchical levels, from gene expression to physiology under dynamic environmental conditions. Key terms framing metabolic regulation as an engineering problem are defined in *Text Box* 1.

One of the major examples of a control system governing plant metabolism is the circadian clock, the manipulation of which could have a beneficial effect on agricultural traits (Bendix et al. 2015). However, in the context of control discussed here, we focus on the fundamental building blocks of regulatory networks and refer the reader to Farré and Weise (2012); Ogasawara et al. (2025).

### Metabolic control analysis and network motifs

Control concepts have long been used to explain metabolic homeostasis (Cannon 1929) and distributed regulation, notably through metabolic control analysis (MCA) (Heinrich and Rapoport 1973; Kacser and Burns 1973; Kacser et al. 1995; Heinrich and Schuster 2012; Klipp et al. 2016; Mazat 2023; Fell et al. 2024). In plants, MCA contributed to a quantitative understanding of sugar metabolism and photosynthesis (Rees and Hill 1994; Uys et al. 2007; Matuszyńska et al. 2019; Saadat et al. 2021; Nies et al. 2023), challenging classical notions, such as rate-limiting steps, by quantifying how enzyme perturbations affect biochemical network fluxes via sensitivity coefficients. Control engineering, by contrast, identifies network architectures: feedback, control, and signal processing motifs independent of parameters. These approaches converge when sensitivity coefficients are analyzed under dynamic conditions: Ingalls extended MCA into the frequency domain, characterizing how regulatory responses depend on the timescale of perturbations (Ingalls 2004).

Characterizing network architecture and motifs has substantially advanced understanding of metabolism, gene regulation, hormonal signaling, and protein–protein interactions in plants (Alon 2007; Pritchard and Birch 2011; Hussey et al. 2013; Borrelli et al. 2015; Briones-Moreno et al. 2017; Lavarenne et al. 2018; Vandereyken et al. 2018; Van den Broeck et al. 2020; Losapio et al. 2021). Network motifs influence how biological components, eg metabolites, respond to external signals (Milo et al. 2002, 2004; Alon 2007; Shoval and Alon 2010; Tyson and Novák 2010; Tyson et al. 2019), resembling functional building blocks of engineered control systems. Embedded in larger networks, they detect signals, regulate responses, and stabilize concentrations (compare Tendler et al. 2018), analogous to filters and controllers producing predictable outputs in engineered systems.

At the same time, motif-based interpretations of metabolic dynamics have important limitations. High motif frequency does not imply a specific function (Vázquez et al. 2004; Mazurie et al. 2005; Goemann et al. 2009); motifs may act only under well-defined conditions (Ingram et al. 2006), and their behavior can change in larger networks due to *retroactivity* (Del Vecchio et al. 2008). Thus, only a subset of motifs, under distinct kinetic and structural constraints, can be meaningfully interpreted as metabolic controllers (*Text Box* 1).

Nonetheless, looking ahead, control engineering, MCA, and network theory will likely be more tightly integrated, with MCA providing local sensitivity in larger engineering-style dynamic models of leaves, organs, or whole plants.

### Controllers in plants

Control engineering principles have gained increasing attention in plant science, driven by advances in mathematical modeling and synthetic biology (Ang et al. 2010; Oishi and Klavins 2011; Yordanov et al. 2014; Harris et al. 2015; Briat et al. 2016; Halter et al. 2017; Briat et al. 2018; Chevalier et al. 2019; Guarino et al. 2019; Halter et al. 2019; Samaniego et al. 2019; Alexis et al. 2021; Filo et al. 2022, 2024; Montefusco et al. 2024). Both engineered and biological control systems regulate a process by quantifying the error between the current and desired state, initiating corrective feedback. This raises central questions: *Which aspects of metabolic regulation in plants can be understood as feedback control problems, and what does this imply for their mathematical representation?*

In engineering, proportional, derivative, and integral control form the building blocks of robust regulation (*Text Box* 1, compare Nise 2019). Integral control is of particular biological relevance, providing a mechanistic basis for robust perfect adaptation. Since the discovery of a motif exhibiting integral control in bacterial chemotaxis (Yi et al. 2000), analogous motifs have been identified across biological networks. Interpreting these motifs as controllers shifts modeling focus from individual reactions to network-level structure: *stability, adaptation, and robustness emerge* not from fine-tuned parameters, but *from specific interaction architectures*.

### Example: Single-E controller in metabolism

Waheed et al. classified integral control motifs into single-E and dual-E controllers (Waheed et al. 2022) (*Text Box* 1). In single-E controllers, a controlled variable *A* is maintained near a desired set-point $A_{s}$ by a manipulated variable *E*, whose concentration reflects the integrated deviation from this set-point (Ni et al. 2009; Drengstig et al. 2012). Both *A* and *E* can correspond to metabolites, proteins, or hormones. Importantly, to achieve real integral control, either zeroth-order degradation of the manipulated variable, *E*, or autocatalysis must be present in the controller motif (Ni et al. 2009; Drengstig et al. 2012).

Single-E controllers can support adaptive responses in both non-oscillatory and oscillatory systems, and can be subdivided into inflow and outflow controllers (Thorsen et al. 2013; Fjeld et al. 2017; Risvoll et al. 2017; Ruoff et al. 2019; Thorsen et al. 2019; Grini et al. 2023).

Coordinated inflow and outflow controllers have been proposed to underlie cytosolic nitrate homeostasis in root epidermal cells (Huang et al. 2012). Cytosolic nitrate concentration is determined by five processes: uptake via high-affinity transporters, nitrate-inducible export via low-affinity transporters, vacuolar storage and remobilization, nitrogen assimilation in the chloroplast, and symplastic transport. Under high external nitrate concentration and increased low-affinity transporter-mediated influx, an outflow controller activates. Increased cytosolic nitrate (controlled variable, *A*) activates efflux transporters named EFT (manipulated variable *E*), which remove excess nitrate from the cell. Homeostasis (integral control) is achieved when EFT is removed by a zeroth-order kinetic reaction. Under low external nitrate, inflow controllers operate: transporters or vacuolar release channels (via a manipulated variable, *E*) increase nitrate supply, while cytosolic nitrate itself (controlled variable, *A*) promotes zeroth-order degradation of *E*. Each of the five processes of nitrate metabolism can be related to either inflow or outflow controllers. Their concerted action determines homeostasis.

Iron homeostasis in root cells of non-graminaceous plants has been hypothesized to follow similar controller logic (Agafonov et al. 2016). In aerobic conditions, soil iron is predominantly present as Fe(III). To facilitate uptake, root cells excrete $H^{+}$ via $H^{+}$-ATPases and reduce Fe(III) by a membrane-bound ferric reductase (FRO2) to Fe(II) in the soil. Uptake of Fe(II) (controlled variable *A*) is then mediated by specialized transporters (IRT1) (manipulated variable *E*). Once Fe(II) is in the cell, it can be either stored in the vacuole, used in chloroplasts or mitochondria, or channeled into symplastic transport. An important role for the iron metabolism during deficiency has been attributed to the transcription factor FIT. The combined effects of multiple integral control motifs involving FIT are responsible for a stable cytosolic iron concentration. In iron deficiency, an inflow controller based on negative feedback mediates iron-dependent degradation of IRT1. FIT acts in an auxiliary feedback loop, translating iron concentration levels into IRT1 degradation rates. Under excess iron, storage is regulated by an outflow controller motif that up-regulates the transport of cytosolic iron into the vacuole via tonoplast transporters.

### Example: Dual-E controllers and other control motifs in metabolism

Contrary to single-E controllers, dual-E controllers are not bound by a zeroth-order removal reaction or autocatalysis, allowing higher metabolic flexibility (Briat et al. 2016, 2018). It has been suggested that the brassinosteroid (BR) homeostasis is based on an antithetic control motif (Waheed et al. 2022). Here, BRs (*A*) bind to their receptor, resulting in the accumulation of the transcription factor BZR1 ($E_{1}$) that suppresses the production of BR. The kinase BIN2 uses ATP ($E_{2}$) to phosphorylate BZR1, leading to its proteasomal degradation.

Other integral or proportional-integral motifs have been proposed. Mairet showed that coupling of transcriptional and post-transcriptional regulation can drive molecular adaptation (Mairet 2018). In the proposed level and activity controller, an output (*y*) drives the production of a modulated variable (*x*) and influences its activation ($x^{*}$). As an example, the adaptation of the plastoquinone (PQ) redox state in oxygenic photosynthesis has been examined: PQ redox state causes phosphorylation-dependent redistribution of light-harvesting complexes (LHCII) between photosystems (state transition, phosphorylated and unphosphorylated LHCII equal $x^{*}$ and *x*, respectively), while also regulating Lhcb transcription and thus LHCII production. This combination of transcriptional and post-transcriptional regulation, among other processes, produces adaptive responses in photosynthesis.

Controller interpretations differ substantially in empirical grounding (Table 1). The nitrate and iron homeostasis frameworks are supported by computational simulations that quantitatively reproduce measured mRNA, protein, and metabolite levels in barley root cells and Arabidopsis, respectively (Huang et al. 2012; Agafonov et al. 2016), providing mechanistic validation beyond structural analogy. The brassinosteroid and PQ redox state interpretations remain conceptual: the proposed controller architectures are mathematically consistent and biologically motivated, but the molecular reactions required for integral control have not been independently confirmed *in planta*.

Text Box 1 **Control engineering for metabolic regulation: Key concepts.**

**Table:** 

Concept	Biological Example and Definition	Visual
Controller	**Definition:** A dynamical system that generates control inputs based on the comparison between a desired and the actual system state (error signal). Controllers drive the system to a desired behavior.	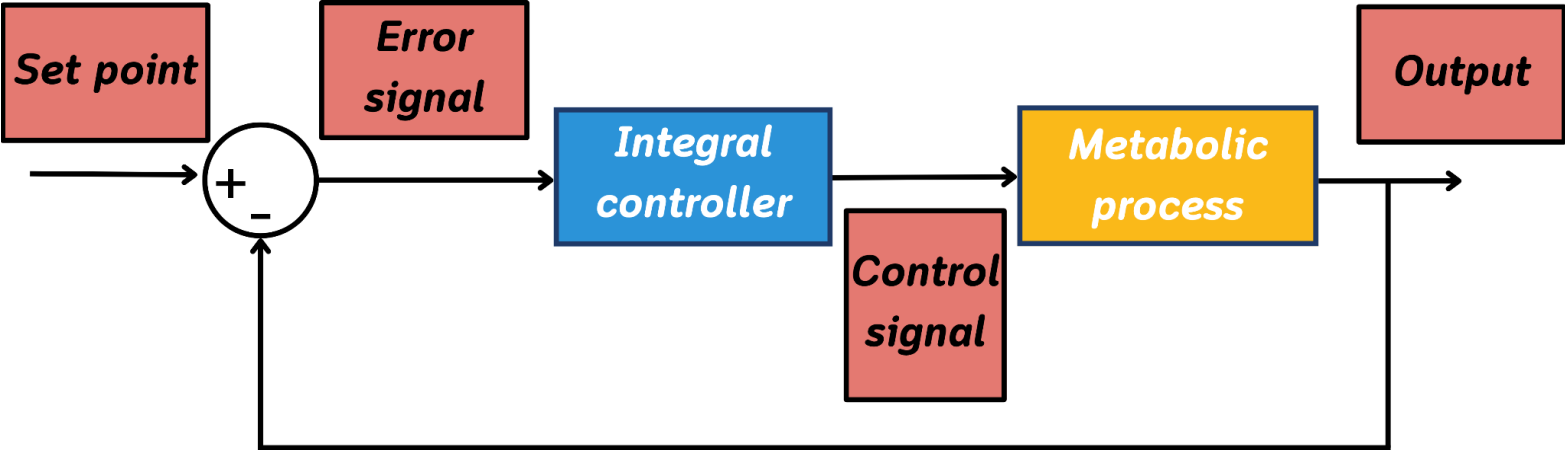
	**Example:** In cytosolic nitrate homeostasis, high nitrate concentration activates EFT that remove excess nitrate. The system continuously measures nitrate levels and adjusts transporter activity accordingly, closing the feedback loop.	
Proportional/ Integral/Derivative (PID) control	**Definition:** Feedback controllers in which the control input is proportional to the integral, derivative, or basic form of the error signal. The controller either provides long-term tracking of a reference signal (integral), better transient and stabilization via anticipatory (derivative), or immediate corrective action (proportional).	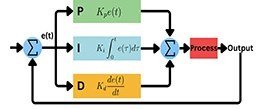
	**Example:** Iron homeostasis in roots: when iron is deficient, the transporter IRT1 accumulates proportionally to the time-integrated iron deficit. This ensures complete correction—the system doesn’t just respond to current error, but tracks cumulative deviation from the setpoint.	
Network motifs	**Definition:** Small, recurring connectivity patterns in biological networks that appear more frequently than expected by chance and often perform specific regulatory functions.	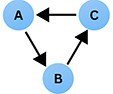
	**Example:** Single-E and dual-E (antithetic) controller motifs are small network patterns (3 to 4 interacting components) that recur across plant metabolism. They detect signals, regulate responses, and stabilize outputs—like functional modules in engineered systems.	
Single-E controller (inflow/outflow)	**Definition:** A control motif with one manipulated variable (E) that regulates a controlled variable (A) toward a setpoint. Outflow controllers remove excess A; inflow controllers supply A when deficient.	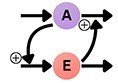
	**Example:** Nitrate homeostasis uses both types: outflow controllers remove excess nitrate via EFT transporters; inflow controllers promote uptake when nitrate is scarce. The manipulated variable (E) must undergo zeroth-order degradation to achieve integral control.	
Antithetic (dual-E) motif	**Definition:** A controller motif with two manipulated variables (${\text{E}}_{1}$, ${\text{E}}_{2}$) that interact and neutralize each other (annihilation). One regulates the controlled variable; the other is influenced by it. Provides metabolic flexibility compared to single-E controllers.	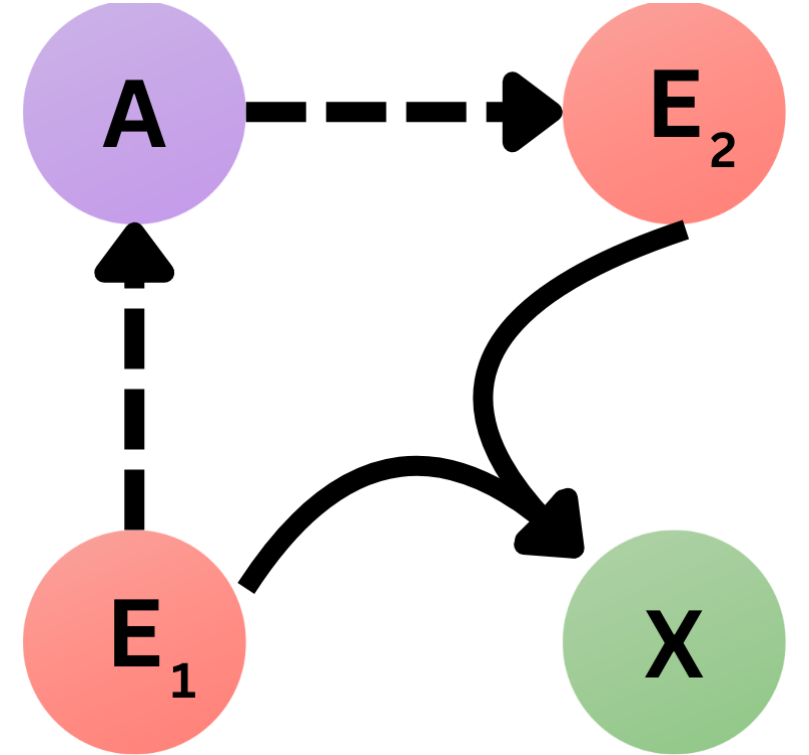
	**Example:** Brassinosteroid homeostasis: BRs bind to receptor producing unphosphorylated BZR1 (${\text{E}}_{1}$), which inhibits BR production. Kinase BIN2 (${\text{E}}_{2}$) phosphorylates BZR1, removing it from the loop. ${\text{E}}_{1}$ and ${\text{E}}_{2}$ annihilate each other, creating integral control without requiring zeroth-order kinetics.	
Robust perfect adaptation	**Definition:** The ability of a system to return to a precise stable output despite sustained disturbances, achieved through integral feedback that eliminates steady-state error.	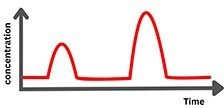
	**Example:** Cytosolic nitrate concentration returns to the same setpoint after disturbances (high external nitrate influx or increased demand), regardless of disturbance magnitude. This homeostasis is “robust”, as it persists across parameter variations.	
Transfer function	**Definition:** A frequency-domain representation of a system’s input–output relationship, obtained via Laplace transformation. Fully characterizes how linear systems respond to inputs at different frequencies.	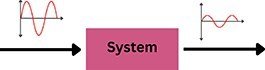
	**Example:** In photosynthesis studies, researchers apply oscillating light at different frequencies and measure chlorophyll fluorescence responses. The transfer function characterizes how photosynthesis “filters” these frequency inputs, revealing regulatory timescales.	
Bode plot	**Definition:** A graphical representation showing how system output magnitude (gain) and phase shift change as a function of input frequency, typically plotted on log scales.	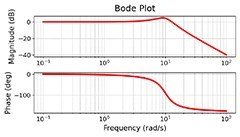
	**Example:** Bode plots of photosynthetic models reveal that the system responds strongly to slow light fluctuations (clouds) but attenuates fast flickers, showing frequency-dependent “filtering” that protects against rapid environmental noise.	
Frequency domain	**Definition:** A representation where system variables are functions of frequency rather than time, enabling analysis of stability and input–output responses across multiple timescales simultaneously.	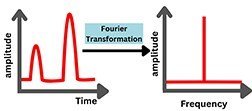
	**Example:** Instead of tracking photosynthetic rate (time domain), frequency-domain analysis reveals which timescales matter most by answering question: *How does the system respond to daily cycles vs. hourly fluctuations vs. second-by-second noise?*	

### Example: Use of control engineering in stomata modeling

Stomata connect leaf interiors with the atmosphere, enabling ${\text{CO}}_{2}$ uptake for photosynthesis while inevitably causing transpiratory water loss. Plants must therefore tightly regulate stomatal aperture and conductance to balance carbon gain with water retention. This balance is complicated by the fact that stomatal conductance responds significantly more slowly to environmental cues than photosynthesis (Lawson and Vialet-Chabrand 2019).

Stomatal movement is regulated by concerted action across multiple levels: hormone networks, guard cell anatomy, ion concentrations, and subsidiary cell water status (Franks and Farquhar 2007; Araújo et al. 2011; Laanemets et al. 2013; Buckley 2017; Lawson and Vialet-Chabrand 2019). Many mechanisms underlying these regulatory processes remain to be revealed.

Modeling stomatal aperture is critical for forecasting climate change effects on land-use efficiency and food supply. Simulations are a well-established tool in stomata research, including empirical, process-based, and optimality approaches (Buckley 2017; Vialet-Chabrand et al. 2017). The latter resembles engineering techniques for optimizing buildings, heating-cooling cycles, or production schedules under multiple constraints. For stomatal conductance, the objective is to maximize photosynthesis while minimizing water loss. Recently, control engineering feedback loops have been explicitly applied to optimality-based stomata models, improving their use in broader land-surface models (Jones et al. 2022). Jones et al. treated stomatal conductance as a control variable in a closed-loop system, using root-finding methods to track the time-varying maximum of leaf photosynthesis weighted by normalized xylem hydraulic conductance.

### Frequency-domain analysis of photosynthetic regulation

Using transfer functions, engineers can fully characterize control systems in the frequency domain (Text Box 1), enabling system responses to be tested across oscillatory inputs. In biology, they can be applied to study a system’s response to external stimuli. Del Vecchio and Murray presented multiple examples of transfer functions and Bode plots for linear and linearized biological models (Del Vecchio and Murray 2015), demonstrating that frequency-domain analyses extend classical input–output investigations and broaden understanding of living system behavior.

Frequency-domain analyses provide a powerful lens to study metabolic dynamics. In photosynthesis research, such analyses have strengthened interest in metabolic regulation and robustness under fluctuating conditions (Nedbal and Březina 2002; Nedbal et al. 2003, 2005). Testing photosynthesis by oscillating light provides a broader picture of the underlying molecular processes, much nearer to natural conditions than pulsed or continuous exposure. Excitation by different light frequencies reveals otherwise obscure nonlinear regulation mechanisms and phenotypes via resonance frequencies and higher harmonics (Nedbal and Lazár 2021).

Frequency-domain analyses have characterized regulatory mechanisms related to the fraction of open reaction centers, the operating frequency of non-photochemical quenching, and the contribution of parallel cyclic electron transport pathways (Lazár et al. 2022; Niu et al. 2023). Mathematical models have been proposed to predict how changes in light amplitude and frequency affect photosynthesis. Fuente et al. developed a model of photosynthesis, which they analyzed using Bode plots (Fuente et al. 2024). Their frequency-domain analysis reveals regulatory properties of photosynthesis that remain hidden under static or step-input experiments, making it a powerful tool for studying plant responses under realistic, fluctuating light.

These frequency-oriented modeling frameworks have inspired data-driven system identification approaches to replicate chlorophyll fluorescence (Bala et al. 2025), allowing direct model extraction from data for control-theoretic analyses. Portilla et al. used the best Linear approximation method combined with linear time-invariant transfer functions to construct a linear parameter-varying representation to extract models of photosynthetic regulation from data based on oscillating light (Portilla et al. 2026).

Together, these approaches, from controller motifs to frequency-domain analysis, reveal that robust metabolic regulation emerges from network architecture rather than parameter fine-tuning. Recognizing homeostasis as arising from specific molecular interactions in single-E or dual-E controller motifs enables targeted experimental questions: Does a metabolite overshoot before returning to steady state? Do enzyme interactions render both partners non-functional, as predicted by an antithetic motif? Can homeostasis be disrupted by manipulating a regulatory intermediate? Grounded in controller theory, such questions inspire experimental designs that steady-state measurements alone cannot resolve. Complementing this, frequency-domain methods expose regulatory timescales invisible to static perturbation experiments, shifting experimental focus from measuring individual reaction rates toward feedback topology and dynamic input–output relationships.

## Structural and mechanical engineering for mechanical stability

Plant form and function obey the same mechanical laws as engineered structures, shaping metabolic activity through light interception, transport efficiency, and mechanosensing (*Text Box 2*). These parallels have driven exchange with structural engineering, yielding tools to model plant structure and predict mechanical responses to environmental forces (Niklas 1992).

Biomechanics, from cell wall micromechanics (Burgert and Dunlop 2011) to wind-induced whole plant movement, demonstrates the power of applying engineering concepts to plant science (Moulia 2013; Geitmann et al. 2019). As outlined in the following paragraphs, plant structural deformations directly inform metabolic activity: leaf inclination governs light interception, and xylem radius limits transport.

### Beam theory allows quantification of plant movement

Beam theory (Euler–Bernoulli or Timoshenko–Ehrenfest), grounded in physics and refined through engineering practice, plays a central role in the design of machines and civil structures (*Text Box* 2).

Many plant organs have high aspect ratios, making them amenable to beam approximations, linking mechanical properties to metabolic outcomes, such as leaf orientation for light interception and, consequently, photosynthetic performance.

How does internal leaf structure determine mechanical behavior and metabolic activity? Modeling iris leaves as sandwich beams, Gibson connected tissue architecture to bending resistances (Gibson et al. 1988). Niklas compared leaf petioles to cantilever beams bending under self-weight and wind load (Niklas 1999), showing how morphology reflects a balance between static, torsion, and bending forces. Leaf position matters for photosynthesis: predicted positions enable simulation of light interception, while mechanical properties determine the extent of foliage movement and thus the light environment in dense stands.

Why do leaves develop particular venation patterns? Treating leaves as thin plates or as discrete beam networks, researchers applied optimality principles similar to those in structural engineering to show that vein patterns maximize rigidity under developmental constraints (Sun et al. 2018; Ronellenfitsch 2021), rigidity that maintains stable leaf orientation against wind and thereby supports consistent light interception for photosynthesis. How do mechanical forces shape development, and how do plants move in response to variable loads? Beam theory applications spanning plant growth, tree trunk swaying, and root buckling reveal how physical forces affect photosynthesis and root exploration in soil (Dean and Long 1986; Coutand et al. 2000; Bizet et al. 2016; Meijer et al. 2019; He et al. 2026). Stem swaying and foliage movement together shape light environments in canopies and photosynthetic biomass production. Micro- and macronutrient uptake through roots is critical for plant metabolism, and predicting root spread using engineering tools could improve crop yield models.

Combined with vibration and fluid mechanics, beam theory addresses how plants respond to fluctuating forces from wind, water, and rain (De Langre 2008; Tadrist et al. 2015; Gardiner et al. 2016; Tadrist et al. 2018). Tadrist et al. showed that wind induces distinct leaf- or branch-dominated movements in trees using image analysis, engineering-based models, and wind-induced drag measurements (Tadrist et al. 2018). They demonstrated that foliage motion arises from two mechanisms that should be integrated into dynamic canopy models to assess photosynthetic performance. Flow-induced drag has also been linked to structural and geometrical features of plants in both terrestrial and aquatic environments, combining plant biology with fluid mechanics (Vogel 1989; Gosselin 2019), enabling prediction of how flowing media alter light interception and photosynthetic activity. Rain-induced oscillations have been studied using beam and vibration theory (Lauderbaugh et al. 2021; Lauderbaugh and Holder 2022), the latter applied in engineering to understand resonance risks and destructive amplification. Plant vibration, a growing field driven by advances in biomechanics, employs vibration engineering to advance understanding of photosynthesis, gaseous exchange, and effects of herbivore attacks in foliage (de Langre 2019; de Langre et al. 2019).

Text Box 2 **Structural and mechanical engineering for mechanical stability: Key concepts.**

**Table:** 

Concept	Biological Example and Definition	Visual
Beam theory	**Definition:** A mathematical framework (Euler–Bernoulli or Timoshenko–Ehrenfest) describing bending, shear, and deflection of slender structural elements under transverse or axial loads, assuming linear elasticity and small deformations.	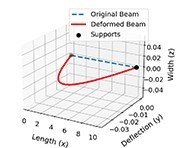
	**Example:** Leaf petioles act as cantilever beams bending under their own weight and wind load. Modeling them this way predicts leaf position and orientation, directly affecting light interception and photosynthetic performance.	
Cantilever beam	**Definition:** A beam fixed at one end and free at the other, subject to loads (gravity, wind) that cause bending with maximum deflection at the free end.	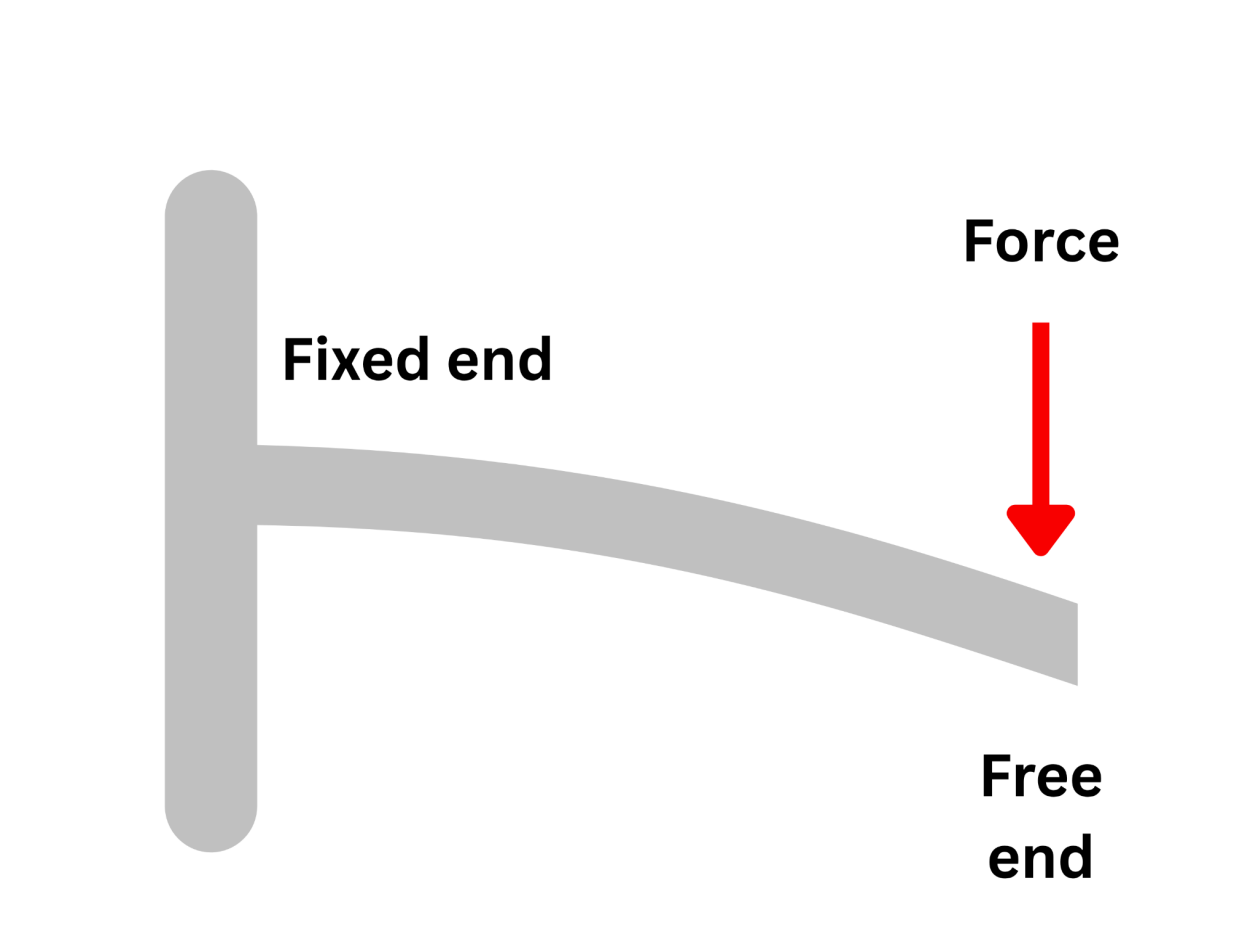
	**Example:** A leaf attached to the stem by its petiole is a cantilever beam—fixed at one end (stem attachment), free at the other (leaf tip). Gravity and wind cause bending that increases with distance from the attachment point.	
Load (static vs. dynamic)	**Definition:** External force applied to a structure. Static loads are constant; dynamic loads vary with time and can induce resonance and vibration.	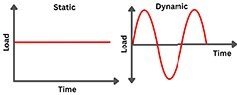
	**Example:** Gravity is a static load (constant over time). Wind and rain are dynamic loads (time-varying), inducing oscillations and vibrations in foliage. Different loads require different mechanical analysis approaches.	
Stress and Strain	**Definition:** Stress: internal forces per unit area within a material (tensile, compressive, or shear). Strain: relative change in size or shape (deformation) resulting from applied stress.	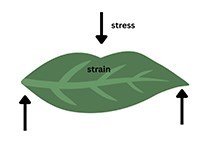
	**Example:** Wind applies external load to leaves, inducing internal stress (force per unit area) in the cell wall. This stress causes strain (deformation): stretching, compressing, or shearing the tissue. Mechanosensors detect this strain and trigger metabolic responses.	
Young’s modulus	**Definition:** A material property quantifying stiffness: the ratio of stress to strain in the elastic regime (E=σ/ε). Higher modulus = stiffer material that resists deformation.	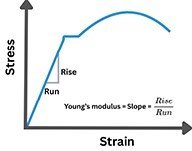
	**Example:** Xylem secondary walls (high modulus ∼10 GPa) resist deformation under negative pressure, while primary cell walls (low modulus ∼100 MPa) allow expansion during growth. These material properties determine mechanical behavior under load.	
Prestress (turgor pressure)	**Definition:** Internal stress intentionally introduced before external loading to improve structural performance. In plants, turgor pressure provides prestress that enhances cell wall rigidity.	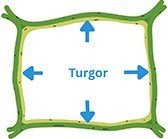
	**Example:** Turgor pressure (cytoplasm/vacuole pushing against cell wall) prestresses the wall, increasing its load-bearing capacity, analogous to prestressed concrete where internal tension improves strength. Loss of turgor compromises structural stability.	
Mechanosensing and transduction	**Definition:** The process by which cells detect mechanical stimuli (stress, strain, pressure) and convert them into biochemical signals that regulate physiological and developmental processes.	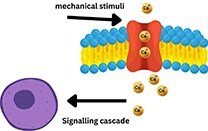
	**Example:** Wind-induced deformation stretches the cytoskeleton and cell wall, activating mechanosensitive ion channels. This triggers ${\text{Ca}}^{2+}$ influx and kinase cascades that alter gene expression, hormone levels, and cell wall reinforcement, translating mechanical stress into metabolic response.	

These examples illustrate that combining engineering predictions of plant position and mechanical properties could sharpen our understanding of photosynthesis, including energy conversion through light interception and biomass production through gaseous exchange. The analysis of plant movement has important consequences for our understanding of photosynthetic regulation as well. Excess light energy that could damage the molecular machinery of photosynthesis is dissipated via non-photochemical quenching (NPQ). However, in fluctuating environments, NPQ relaxation is regularly slower than the change in light conditions, leading the plant to be in a state of prolonged energy dissipation that lowers biomass production. Building models that combine mechanical properties (estimated via beam theory), photosynthesis, and gaseous exchange could help predict new targets for breeding that alleviate the effect of extended NPQ.

Despite its potential, beam theory has limitations in plant applications. Plants comprise composite materials with spatially varying mechanical properties, unlike the elastic, isotropic, homogeneous beams typically assumed in engineering (Dunlop and Fratzl 2010; Niklas and Walker 2021). Beam theory also requires knowledge of material properties, which remain difficult to measure due to the fragility of many plant structures. Integrating ML to predict material properties may help address this challenge (Kibrete et al. 2023), enabling more accurate models of plant biomechanics. Not all beam-theory applications discussed above carry equivalent empirical support. The petiole cantilever, iris leaf sandwich beam, stem deformation, and root buckling models (Dean and Long 1986; Gibson et al. 1988; Niklas 1999; Coutand et al. 2000; Meijer et al. 2019; He et al. 2026) have been validated against experimental measurements of leaf position, flexural rigidity, or stem form in plants; by contrast, the following prestressed-concrete analogy for turgor pressure (Dolan and Hamilton 2019) and the road-design analogy for cytoskeletal geometry (Nasrin et al. 2020, 2021) are instructive but remain qualitative comparisons without direct quantitative confirmation *in planta* (again, Table 1).

### Cytoskeleton and biomembranes—scaffolds of the cell

Structural and mechanical engineering concepts extend to the cellular level, where mechanical properties constrain metabolic organization. The arrangement of organelles, vesicle routing, and accessibility of reaction sites all depend on the cell’s mechanical scaffolding. What determines cell stability when, unlike in animal cells, the cytoskeleton plays only a minimal structural role? This question can be approached through engineering analogies combining internal pressure with external containment.

The plant cytoskeleton, a network of actin filaments and microtubules, is crucial for cell growth and development (Gutierrez et al. 2009; Woodhouse and Goldstein 2013; Zhang et al. 2019). Unlike in animal cells, where it provides structural integrity, in plant cells, it primarily drives intracellular transport and mechanoperception. Actin cables route vesicles to target compartments, position organelles at membrane contact sites, and their mechanical properties affect the speed and directionality of motor-driven cargo. Notably, chloroplasts move along actin filaments in response to light, relocating away from high-intensity regions to prevent photodamage.

Plant structural stability stems from vacuole and cytoplasm pressure against the cell wall (Codjoe et al. 2022), a cellulose matrix composite whose mechanical properties respond to environmental cues (Hamant and Traas 2010; Shteinberg et al. 2024). By constraining cell volume, the cell wall restricts metabolic processes and demands precise compartmentalization. This pressurized stability is analogous to prestressed concrete: steel tendons are stretched, and concrete is poured over them; upon curing and release, tendon contraction induces compressive stress, increasing load-bearing capacity and improving stability (Dolan and Hamilton 2019).

How does cytoskeletal geometry influence intracellular transport and metabolic activity? Measurements and beam theory have revealed the mechanical properties of actin filaments and microtubules (Gittes et al. 1993; Satcher and Dewey 1996; Kis et al. 2002; Pegoraro et al. 2017), from which filament bending and curvature can be inferred. These filament deformations directly affect motor-driven vesicle transport and, in turn, metabolic efficiency (Nasrin et al. 2020, 2021). This is much like road design in civil engineering, where curves and bumps guide the motion of vehicles through deliberate geometric placement.

Freed from structural support, the plant cytoskeleton shifted toward processing mechanical stimuli (Monshausen and Haswell 2013; Hernández-Hernández et al. 2014; Hamant et al. 2019), which influence cell proliferation and growth by altering metabolic processes. When osmotic changes perturb the balance between turgor pressure and cell wall stiffness, cells detect the disturbance and restore mechanostasis through growth, osmoregulation, or wall strengthening (Codjoe et al. 2022). How does the cytoskeleton translate mechanical stimuli into intracellular signals? The cytoskeleton functions as part of an integrated mechanosensing network, including the cell wall and mechanosensitive proteins. These mechanosensors, ion channels, or receptor-like kinases (Cox et al. 2017) activate ${\text{Ca}}^{2+}$ influx and kinase cascades, translating mechanical stimuli into metabolic or genetic responses, including cytoskeletal reorganization, altered vesicle transport, and hormonal regulation (Hansen et al. 2025). Microtubules align with maximal tensile stress, steering cellulose synthase to sites needing mechanical reinforcement (Sampathkumar et al. 2014). Cytoskeletal-mediated mechanoperception is also critical for morphogenesis: developmental tissue responds to mechanical stress and strain, with these forces guiding differentiation (Hamant et al. 2008; Hamant and Traas 2010). A recurring mechanical input can elicit thigmomorphogenesis, an adaptive morphogenetic response characterized by reduced elongation and increased thickening of stems and other organs (Jaffe 1973). These anatomical changes alter canopy architecture, modulate light interception and self-shading, and thereby influence photosynthetic performance and biomass allocation (Chehab et al. 2009; Coutand and Mitchell 2016).

Text Box 3 **Fluid Dynamics for Metabolic Transport: Key Concepts.**

**Table:** 

Concept	Biological Example and Definition	Visual
Reynolds number (Re)	**Definition:** A dimensionless number Re=ρvD/μ comparing inertial forces to viscous forces. Low Re (≪1) = viscosity-dominated laminar flow; high Re (>2,000) = inertia-dominated turbulent flow.	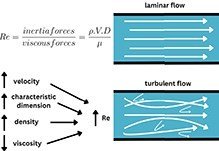
	**Example:** In xylem vessels and phloem sieve elements, Re≪1 (viscous forces dominate), creating smooth laminar flow. High-Re turbulent flow is essentially absent in plant vascular systems.	
Laminar flow	**Definition:** Flow in smooth, parallel layers with no mixing between layers, characteristic of low Reynolds number regimes.	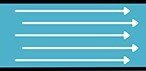
	**Example:** Xylem sap flows in smooth parallel layers along vessel walls with minimal mixing between layers. Cytoplasmic streaming also exhibits laminar flow patterns.	
Navier–Stokes equations	**Definition:** Fundamental equations describing fluid motion that integrate conservation of mass, momentum, and energy. Simplified forms (Stokes flow, lubrication theory) apply to low-Re biological flows.	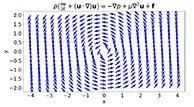
	**Example:** Used in CFD models to simulate cytoplasmic streaming, phloem transport, and xylem flow patterns.	
Hagen–Poiseuille law	**Definition:** For laminar flow through cylindrical conduits: flow rate $Q\propto r^{4}\Delta P/(\mu L)$, where *r* = radius, ΔP = pressure difference, *μ* = viscosity, *L* = length.	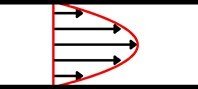
	**Example:** Doubling xylem vessel radius increases flow rate 16-fold ($r^{4}$ dependence), explaining why wider vessels dramatically improve hydraulic conductivity but also increase cavitation risk.	
Cohesion-tension theory	**Definition:** The mechanism driving xylem water transport: evaporation at leaf surfaces creates negative pressure that, due to water cohesion, pulls the water column upward from roots.	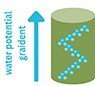
	**Example:** Water evaporation in leaves creates capillary forces generating negative pressure (tension) that pulls water upward through xylem without active pumping, but risks cavitation.	
Münch pressure-flow mechanism	**Definition:** The osmotic pump driving phloem transport: solute loading creates pressure gradients that drive mass flow from source to sink tissues.	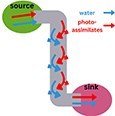
	**Example:** Sugar loading in leaf phloem lowers water potential, drawing water from xylem and creating high turgor pressure. The pressure gradient between source (leaf) and sink (roots) drives bulk flow of photosynthates.	
Cytoplasmic streaming	**Definition:** Active intracellular fluid circulation driven by motor proteins that creates mass flow to enhance mixing and distribution beyond diffusion-limited transport.	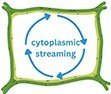
	**Example:** Myosin-XI motors transport ER along actin filaments, generating bulk cytoplasmic flow that distributes metabolites, enzymes, and organelles more rapidly than diffusion alone, overcoming size-based diffusion limitations in large cells.	
IC resistance (integrated circuit)	**Definition:** Internal resistance within a circuit. The opposition to fluid flow through a conduit, analogous to electrical resistance. Higher resistance requires a greater pressure difference to maintain the same flow rate.	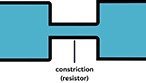
	**Example:** In phloem, sieve plates and cell lumen contribute nearly equally to total resistance. Narrower conduits and sieve plate pores increase resistance, limiting transport rates.	
Darcy’s Law	**Definition:** Fluid flow through a porous medium.	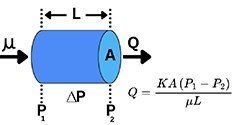
	**Example:** Soil water is taken up by roots and subsequently transported throughout the plant, where its movement can be modeled as fluid flow within a hydraulic system.	
Young–Laplace equation	**Definition:** The Young–Laplace equation relates the pressure difference across a curved liquid–air interface to its curvature and the liquid’s surface tension.	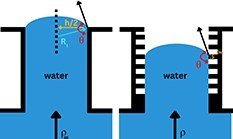
	**Example:** The driving force behind water transport in the xylem is transpiration. As water evaporates from liquid surfaces into the air, the remaining water forms curved surfaces (menisci). As these surfaces become more curved, the radius decreases, which lowers the pressure below the air pressure. This creates tension (negative pressure) that pulls water upwards through the xylem.	
Bernoulli’s principle	**Definition:** Increasing fluid speed is accompanied with a decrease in pressure (steady, incompressible, non-viscous flow along streamlines).	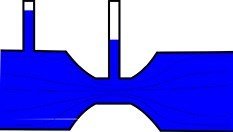
	**Example:** While water flows through the xylem, based on the cohesion-tension mechanisms, variations in vessel diameter can lead to changes in flow velocity and pressure.	

Biomembranes balance structural integrity with controlled transport and dynamic remodeling. Their thin but extended structure invites comparison with thin plates in engineering, opening questions addressable with engineering tools.

What determines membrane integrity while enabling dynamic compartmentalization? Membranes are complex viscoelastic structures that compartmentalize cellular space and enable the coexistence of biochemical pathways. Variations in lipid and protein compositions give rise to distinct mechanical properties (Jacquot et al. 2014) analogous to material parameters in engineered thin structures. Membrane tension in particular plays a key role in maintaining organelle integrity and preventing compartment fusion (Upadhyaya and Sheetz 2004).

What controls membrane shape at the molecular level? Lipid geometry (eg cones or cylinders), domain formation, and protein integration generate bending stress, asymmetries, and tension that collectively influence membrane curvature and undulations (Sprong et al. 2001; Lipowsky et al. 1998), like how heterogeneity, inclusions, and prestress shape engineered plates and shells.

Can engineering theories predict membrane behavior? The Canham-Helfrich theory for biomembranes and the classical thin-plate theory both analyze the bending energy of thin surfaces. In both frameworks, deformation is described through curvature-dependent energy functionals, enabling prediction of equilibrium shapes and the amplitudes of undulation modes (HW et al. 2002). However, the two theories operate on different scales where different forces dominate, requiring distinct terms and assumptions in their governing equations.

Spatial organization of thylakoid membranes has been observed in both plants and cyanobacteria (Kirchhoff 2008; Kaňa et al. 2023). Chloroplasts contain an intricate system of stacked (grana) and unstacked (lamellae) membrane regions, within which photosynthetic complexes are distributed across domains to regulate photosynthesis. Analogous spatial organization has been observed along and within cyanobacterial thylakoid membrane sheets. How and why this organization arises remains an open question, to which engineering concepts such as thin-shell bending could contribute.

The cytoskeleton, cell wall, and biomembranes form an integrated mechanical framework that maintains cell stability, enables transport, and shapes metabolic activity through compartmentalization, intracellular transport, and mechanosensing.

## Fluid dynamics: Transport constraints on metabolic function

The mechanical properties of plant structures, vessel radius, cell wall elasticity, and cytoskeletal geometry simultaneously constrain fluid flows on which metabolism depends. Understanding how fluids move through and within plants is, therefore, the natural complement to the structural analysis, forming the third engineering domain considered in this review. Key processes include xylem water transport, phloem assimilate distribution, and cytoplasmic streaming.

In classical engineering, fluid dynamics operates in the high-Reynolds-number regime, informing material selection under wind- and water-flow-mediated forces (Launder and Spalding 1974; Idrissi et al. 2025). The rise of microfluidics has shifted focus toward viscosity-dominated, low-Reynolds-number flows, forging new connections to biology, where small scales and crowded environments make low Reynold numbers the norm (Jensen et al. 2012; Evans and Morris 2017; Strickland et al. 2017). These have opened avenues for applying computational fluid dynamics tools to model fluid motion in cells or whole organisms, predicting metabolite distribution throughout biological systems. Knowledge of flow patterns is, therefore, increasingly recognized for understanding metabolic processes, as the *spatial distribution of metabolites can determine rates, activity, and cellular function*.

### Understanding cytoplasmic streaming and long-distance transport

Intracellular cargo transport along the cytoskeleton drives cytoplasmic streaming in algal and plant cells (Shelley 2024), helping overcome diffusion limitations hindering metabolism (Hochachka 1999). In plants, motor proteins such as myosin-XI carry the endoplasmic reticulum along actin filaments, generating cytoplasmic flow that evenly distributes and mixes cellular contents, which becomes critical as cell size increases and diffusion alone becomes insufficient. Cytoplasmic streaming serves as the cell’s circulation system, distributing metabolites, enzymes, and organelles (Dodonova and Bulychev 2012; Lu and Gelfand 2023), much like a heating, ventilation, and air conditioning (HVAC) system regulates airflow. Both overcome diffusion limitations through active transport: HVAC systems use fans and ducts; cytoplasmic streaming uses motor-driven flow.

Fluid movement also governs long-distance transport in plants. The vascular system, comprising xylem and phloem, two distinct “pipeline” networks, transports water from the soil throughout the plant and distributes assimilates from the source (leaves) to sink tissues (De Schepper et al. 2013; Sack and Scoffoni 2013; Venturas et al. 2017; Jensen 2018; Katifori 2018), analogous to plumbing and HVAC pipeline networks. Indeed, fluid flow through plant vasculature is often described by equations from fluid dynamics and engineering disciplines that address the distribution of liquids (Jensen 2018). Bernoulli’s principle, Darcy’s law, Navier–Stokes equation, the Young–Laplace equation, and Hagen–Poiseuille law, among others, are frequently found in mathematical treatments of xylem or phloem transport (Vesala et al. 2003; Hölttä et al. 2006, 2009; Jensen et al. 2012; Mammeri and Sellier 2017) (*Text Box 3*). Xylem vasculature has been compared to a porous medium, like a wick (Sperry 2011). According to the cohesion-tension theory, evaporation in leaf water channels generates capillary forces that create negative pressure, driving bulk water movement and allowing plants to extract water without active pumping (Venturas et al. 2017). However, this negative pressure also risks capillary failure, cavitation, or implosion (Sperry 2011; Venturas et al. 2017), necessitating active regulation (Nardini et al. 2011; Kaack et al. 2021). Interestingly, analogous pressure-induced damage occurs in engineered plumbing near pumps, bends, or valves.

Phloem transport is less well understood than the movement of water through the xylem. The accepted model is the Münch theory, based on an osmotic pump/pressure-flow mechanism, transporting sugar molecules (Knoblauch and Peters 2010; Knoblauch and Oparka 2012; Jensen 2018; Konrad and Roth-Nebelsick 2023). These transported sugar molecules are products of the CBB cycle, which is fueled by ATP and NADPH generated through photosynthesis, mainly occurring in leaves. Beginning in the leaves, sugars are loaded into the sieve elements by active or passive symplastic or apoplastic transport (De Schepper et al. 2013), which results in a lower water potential and thus an inflow of water from the xylem, generating higher turgor pressure. The rising pressure gradient between source and sink drives bulk mass flow. Unlike xylem, an engineering comparison for phloem is less straightforward, as transport occurs through living tissue rather than passive conduits. However, recently, it has been shown that the elasticity of sieve element cell walls may be critical for understanding fluid flow in the phloem system at low Reynolds numbers (Nakad et al. 2023). This directly relates to active research in mechanical engineering on microfluidic devices, in which low-Reynolds-number hydrodynamic and fluid-structure interaction in confined flows play a critical role (Anand and Christov 2021). The modeling of fluid transport via xylem and phloem directly relates to predictions of photosynthesis. Recently, Joshi et al. developed an analytical trait-based optimality model combining hydraulics throughout plants with photosynthesis to predict stomata aperture and acclimation to various climatic conditions (Joshi et al. 2022). Modeling results show good agreement with experimentally measured data.

Modeling HVAC systems, pipe networks, cytoplasmic streaming, and xylem/phloem transport is vital for their analysis. Simulation is frequently based on computational fluid dynamics (CFD) in technical and biological systems. Based on the Navier–Stokes equations, which integrate the conservation of mass, momentum, and energy, CFD predicts fluid flow. Combined with artificial intelligence to predict the system’s properties, CFD and exploratory simulations accelerate the engineering design process tremendously (D’Aquilio et al. 2023). CFD for biological phenomena draws on numerical insights from fluid flow in technical systems. Goldstein et al. (2008) used Stokes flow in a low Reynolds-number regime to model cytoplasmic streaming. In Htet and Lauga (2025), lubrication theory, which approximates the Navier–Stokes equation, is applied as a framework for modeling cytoplasmic flow in elongated cells of four different organisms. Jensen et al. used CFD to investigate the fluid flow around phloem sieve plates, showing that sieve plate and cell lumen resistance contribute almost equally to the overall hydraulic resistance to phloem transport (Jensen et al. 2012). The Lattice–Boltzmann algorithm has been applied to the interaction between cytoplasmic fluid flow and spherical particles, such as vesicles (Wolff et al. 2012). Computational fluid dynamics has been applied to model xylem water transport, dissecting resistance partitioning between perforation plates, pit structures, and smooth vessels in *Jatropha curcas* (Xu et al. 2020). Xu et al. combined precise structural measurements with a three-dimensional xylem model of fluid flow based on Navier–Stokes equations, extracting each xylem structure’s contribution to flow resistance. Through further variation of inner vessel diameters, pit depths, heights, and widths of the perforation plates, the authors were able to correlate parameters with flow rates inside vessels, pinpointing how fluid flow can be increased or decreased.

Beyond CFD, Darcy’s law of plug flow has been used to model coupled water and solute transport through the xylem and phloem (Sakurai and Miklavcic 2021), incorporating diffusive effects and hydraulic pressure between plant nodes. By varying leaf shape, Sakurai and Miklavcic deduced that geometry has the largest influence on the hydraulic pressure distribution in the xylem. The narrower the leaf, the lower the area-average water pressure, laying a foundation for exploring leaf shape and vascular architecture.

The transport and CFD models discussed here differ substantially in empirical grounding. The xylem CFD analysis by Xu et al. (2020) and the phloem sieve-plate resistance study by Jensen et al. (2012) are computationally validated but rely on steady-state conditions and specific vessel geometries that are technically difficult to obtain at scale. Phloem models remain further limited by poorly constrained parameters. Osmotic coefficients, membrane permeabilities, and sieve-plate elasticities are condition-dependent and rarely measured simultaneously, while cytoplasmic streaming models (Goldstein et al. 2008; Htet and Lauga 2025) have so far been applied to algal or simplified cells and have not produced quantitative predictions of metabolic flux in higher plant tissues.

## Cross-domain synthesis: Stomatal control of carbon gain under fluctuating environments

**Figure 2:**
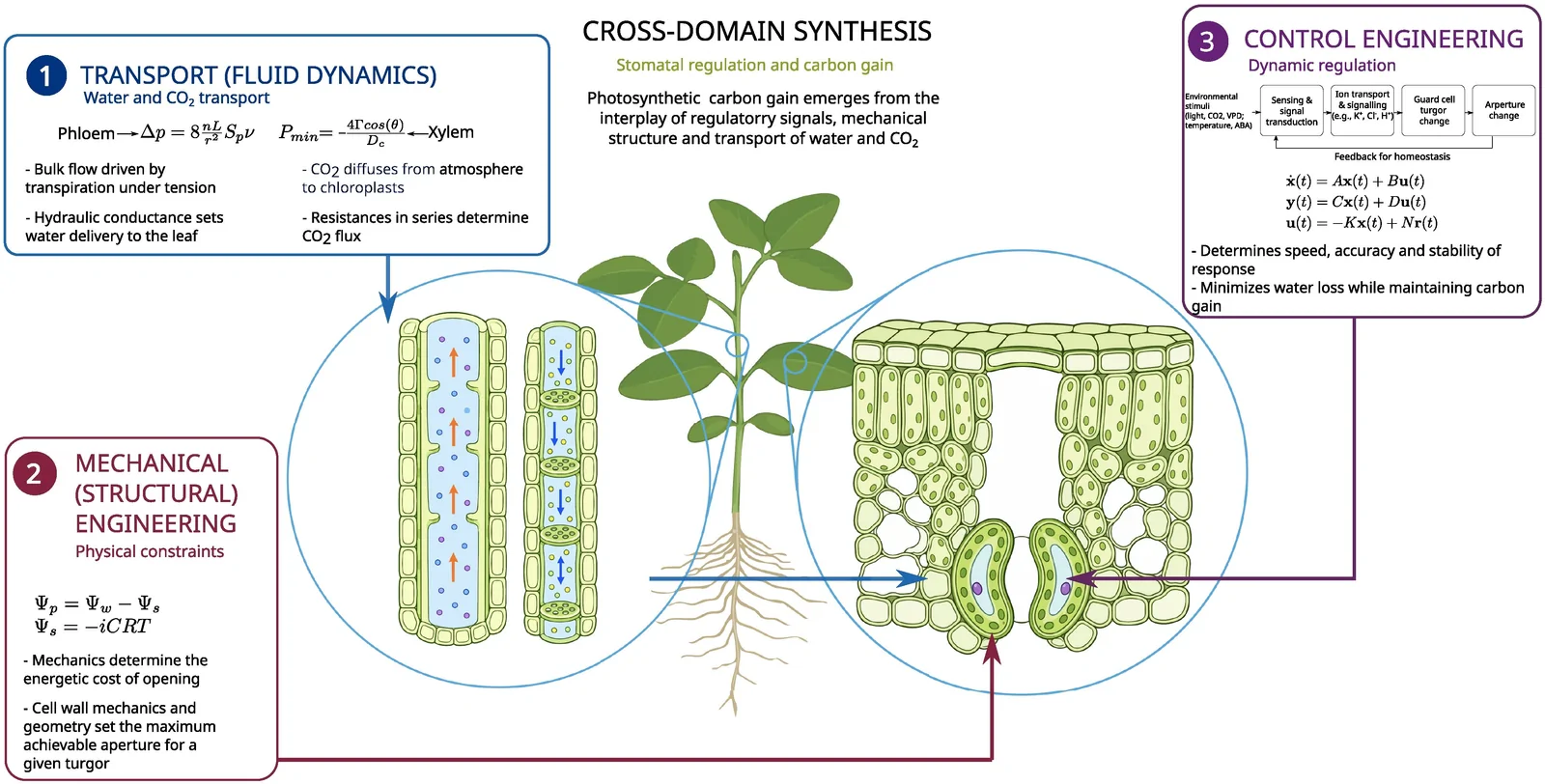
Stomatal regulation and carbon gain using formalisms from engineering domains. Fluid dynamics governs the hydraulic conductance of the xylem-to-guard-cell pathway, constraining how rapidly the aperture can change in response to biochemical signals, with characteristic timescales of approximately 1 to 10 min. As a first approximation, phloem solute transport via pressure difference can be described by Darcy’s law (*η* sap viscosity, L total transport distance, $S_{p}$ resistance through sieve pores, flow speed *ν*). Xylem water transport is driven by negative water pressure. The most negative pressure that a meniscus in a water capillary can generate is described by the depicted formula (*Γ* surface tension, *θ* contact angle between liquid and capillary surface, $D_{c}$ capillary diameter). See Venturas et al. (2017); Jensen (2018) for more details. Structural mechanics determines the guard cell wall elasticity and geometry that define the admissible aperture range and actuator gain; these are quasi-static structural parameters that bound the solutions available to the stomatal control system. Turgor pressure links biochemical signals and structural deformation ($\Phi_{p}$ turgor pressure, $\Phi_{w}$ water potential, $\Phi_{s}$ solute potential, *i* ionization constant, *C* solute concentration, *R* gas constant, *T* temperature). Control engineering describes the biochemical signaling cascade (ion transport, ABA networks) that sets guard cell turgor and the aperture set-point, operating on timescales of approximately 10 to 30 min. State space models can be used to describe the feedback dynamics (*A* system matrix, *B* input matrix, *C* output matrix, *D* feedthrough matrix, *K* feedback, *N* reference signal matrix, x(t) state vector, u(t) control input vector, y(t) output vector, r(t) reference input). The coupling between the domains is constitutive: guard cell turgor integrates regulatory inputs and hydraulic state simultaneously; the resulting aperture determines ${\text{CO}}_{2}$ flux and thereby photosynthetic carbon gain.

To illustrate how the three engineering domains reviewed above can be brought together toward the unified mechanistic framework described in the Introduction, we use stomatal regulation of carbon gain as an integrative example (Fig. 2). It is the natural test case for bridging these layers, as stomatal movement in fluctuating environments is governed by a multi-scale regulatory system.

First, stomatal regulation combined with photosynthesis constitutes an optimization problem that aims to maximize carbon assimilation while minimizing water loss, placing stomatal dynamics within the class of constrained optimal control problems with state-dependent actuation limits (Jones et al. 2022).

Second, at the tissue level, guard cell deformation determines aperture. Researchers modeled turgor-driven deformation by treating stomata as thin-walled tori with elastic composite cell walls (Cooke et al. 1976; Woolfenden et al. 2017). Guard cell bending, elongation, and cell interactions were modeled using beam theory (Yi et al. 2019). Guard cell turgor, determined by ion-transport and signaling networks, serves as the primary link between biochemical regulation and mechanical deformation, a linkage integrated across several computational platforms (Chen et al. 2012; Hills et al. 2012; Cong et al, 2024).

In parallel, water flux required to drive guard cell deformations is governed by hydraulic transport along the xylem–mesophyll–epidermal continuum, introducing an additional dynamical constraint on response speed (Tardieu and Davies 1993; Dewar 2002).

Bringing these three layers together could explain a phenomenon that, so far, neither framework predicts alone: the characteristic lag of stomatal conductance behind photosynthetic capacity under fluctuating light (Lawson and Vialet-Chabrand 2019). The integrated framework could therefore identify which layer (signaling kinetics, hydraulic conductance, or wall elasticity) represents the dominant bottleneck under a given environmental regime and provide a quantitative basis for targeting these properties in breeding or synthetic biology approaches aimed at improving light-use efficiency.

Looking forward, ion transport, turgor, shape changes, and water transport could be modeled by a system of coupled differential equations in which stomatal aperture feeds back into carbon assimilation, defining the control target. To our knowledge, the approach of Joshi et al. (2022) most closely approximates this vision, coupling stomatal movement with biochemical regulation.

Nonetheless, formulating stomatal dynamics as a coupled system of differential equations remains challenging due to parameter identifiability across scales. While the governing equations follow from physical and biochemical principles, key parameters remain condition-dependent or experimentally inaccessible. Hybrid modeling strategies discussed in the next section address this by embedding data-driven components into mechanistic models. Rather than replacing structure with flexibility, these approaches retain the solution space defined by control, mechanical, and transport constraints, while enabling effective parameterization.

## Computational engineering: Hybrid modeling and machine learning for metabolic dynamics

As the mechanistic models grow in complexity, incorporating multi-scale regulation, spatial heterogeneity, and coupled physical processes, they encounter practical limits: parameter spaces become intractable, measurements may be incomplete, and analytical solutions unavailable. This motivates consideration of complementary computational approaches that learn patterns from data rather than deriving behavior from first principles. ML and artificial intelligence represent a fundamentally different engineering paradigm: rather than deriving models from physical principles, patterns are being discovered from the data, and relationships are inferred from them. While this approach, in most of the current cases, leads to the creation of a so-called “black-box” model that lacks mechanistic insight and thus the blueprint needed for rational engineering, control, and optimization, recent advances in physics-informed ML demonstrate that hybrid methods can combine the interpretability of physics with the flexibility of learning. Physics-informed neural networks (PINNs) embed known physical laws directly into neural network architectures, ensuring learned models respect fundamental constraints like mass conservation, energy balance, or thermodynamic limits (Raissi et al. 2019). For plants, this offers a powerful approach when partial knowledge exists, but complete mechanistic models of metabolic pathways are intractable. Consider photosynthesis and subcompartmental sugar metabolism. Biochemical reaction kinetics are well-established, but dynamic regulation involves complex feedback that resists simple formulation. A PINN could learn these regulatory dynamics from metabolomic timecourse data, while enforcing mass conservation effectively, proposing previously “missing” terms in mechanistic models. Similarly, universal differential equations (Rackauckas et al. 2020) replace unknown components of mechanistic models with neural networks, creating hybrid models. What may seem computationally straightforward (learning dynamics from data) comes with classical challenges that are actively addressed by engineering, like inferring model parameters or internal states from noisy measurements using Kalman filters (Zhang and Su 2002; Khodarahmi and Maihami 2023). ML offers new strategies through simulations to tackle these problems (Wendering and Nikoloski 2026). Estimating photosynthesis model parameters from fluorescence transients currently requires iterative fitting; a trained neural network could provide instant parameter estimates, enabling high-throughput phenotyping (Li et al. 2016). A similar approach was recently used to estimate parameters of a large-scale kinetic model of ${\text{C}}_{4}$ photosynthesis. Wendering et al. trained a neural network based on photosynthesis response curves, using a surrogate model to speed up training. Applied to 68 maize genotypes across two seasons, the network predicted parameter sets that allowed the identification of key limitations to photosynthetic efficiency that were validated using simulations (Wendering et al. n.d.). The same approach applies to structural mechanics: predicting bending moduli and elastic properties of plant composite materials from anatomical features using ML (Kibrete et al. 2023) would enable high-throughput parameterization of beam-theoretic models, circumventing the laborious in vivo measurements currently required. Beyond parameter estimation, ML has shown growing potential for system identification, supporting the discovery of dynamical models from time-series data. Symbolic regression algorithms search for interpretable mathematical equations that fit experimental data, potentially rediscovering known physical laws or revealing unexpected relationships (Bartlett et al. 2025). These approaches bridge data-driven discovery and mechanistic modeling, offering pathways to extract interpretable equations from complex experimental data and the creation of *digital twins*. Originally developed for aerospace and manufacturing, digital twins enable prediction, optimization, and control by simulating alternative scenarios faster than real-time. They are a virtual replica of a physical biological system, continuously updated with real-time data to mirror its actual counterpart (Attaran and Celik 2023). Early plant digital twins demonstrate feasibility: tomato growth models integrated with greenhouse sensors and Kalman filtering for state estimation enable model predictive control of climate, optimizing yield while minimizing resource use (Smoleňová et al. 2025) (For reviews of the digital twin concept for food supply chains and greenhouse horticulture, see Defraeye et al. 2021; Ariesen-Verschuur et al. 2022). Yet, despite the advancements, applying computational methods to plant systems faces significant challenges, ranging from data scarcity to insufficient plant data across genotypes, environments, and developmental stages, as well as a lack of software infrastructure that supports the modular construction, simulation, and interrogation of hybrid dynamical models. The emergence of open-source environments for hybrid modeling in life sciences, such as HybridML (Merkelbach et al. 2022) or MxlPy (van Aalst et al. 2025), is the prerequisite for applying such approaches. In fluid dynamics, surrogate ML models replacing computationally expensive CFD simulations have already been demonstrated in engineering design (D’Aquilio et al. 2023). Applied to xylem vessel geometry datasets such as those assembled by Xu et al. (2020), such surrogates could enable resistance partitioning analyses at a scale currently inaccessible to full numerical simulation. Nonetheless, the convergence of physics-based modeling, ML, and adaptive estimation methods represents a natural evolution of quantitative plant science, with *hybrid frameworks balancing the strengths of mechanistic insight and data-driven flexibility*. Recent work on mechanistic modeling of plant respiration demonstrates that explicitly representing enzyme kinetics and network constraints enables a transition from phenomenological descriptions to models capable of informing rational metabolic engineering (Wendering and Nikoloski 2023). This example illustrates why mechanistic structure is indispensable for biological interpretation and intervention. Given that qualitative system behavior depends critically on kinetic details that purely data-driven approaches may overlook, ML will likely be most powerful when combined with physically grounded models rather than applied in isolation. Multi-stressor robustness is precisely the type of problem for which such hybrid approaches, drawing on control engineering and structural reliability theory, are particularly well suited.

## A critical assessment: SWOT analysis for applying engineering methodologies in plant metabolism

**Figure 3:**
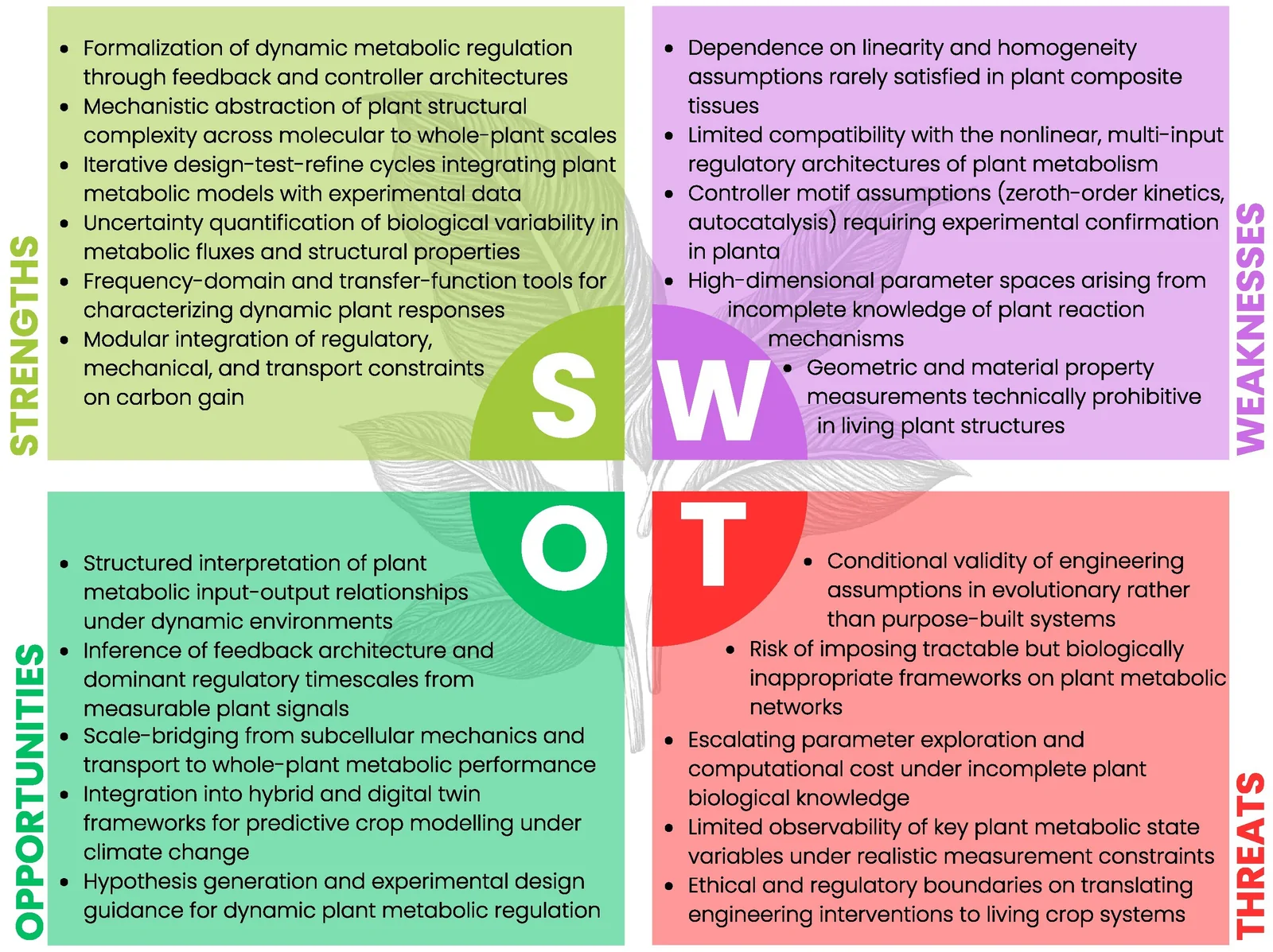
Strategic analysis of engineering frameworks for plant metabolism. SWOT of engineering methodologies for modeling plant metabolic processes and integrating multi-scale biological data.

Having established how control, mechanical, and transport engineering frameworks characterize plant metabolism from molecular homeostasis to whole-plant carbon gain, we now critically assess their collective strengths, weaknesses, opportunities, and threats (SWOT), distinguishing established biological insight from conceptual proposals (Fig. 3). Bringing these frameworks together highlights that plant metabolism operates under layered constraints: stoichiometric (addressed by genome-scale models and flux balance analysis), kinetic (addressed by mechanistic ordinary differential equations models), and physical (addressed by the control, mechanical, and transport frameworks presented here). These constraint layers are not alternatives but operate simultaneously: metabolic fluxes must satisfy mass balance, obey reaction kinetics, and *respect physical limits on information processing, force balance, and transport capacity*.

The **strengths** of engineering methodologies lie in their ability to abstract complex and formalize the layered complexity of plant metabolism. Plants are difficult to analyze within a unified mathematical framework, precisely because they are multilayered and nonlinearly regulated across timescales ranging from milliseconds to seasons. However, conceptualizing petioles as beams, xylem and phloem as pipe networks, or photoprotective processes as regulatory circuits and feedback controllers enables quantitative reasoning even in the presence of uncertainty and biological heterogeneity. Moreover, engineering disciplines have developed robust frameworks for uncertainty quantification and confidence assessment, which are particularly valuable in biological systems, given the well-documented variability between individual plants that may itself reflect metabolically meaningful differences. Their iterative design-test-refine cycles, tightly coupling model construction, controlled perturbation, and experimental validation, are becoming increasingly central to quantitative plant biology.

Despite these strengths, engineering and plant biology differ fundamentally in how systems are structured, constrained, and perturbed; **weaknesses** that constrain direct methodological transfer. Many engineering tools rely on simplifying assumptions, such as linearity, well-defined system boundaries, stable operating regimes, or noise within known limits, to remain analytically manageable. Plant metabolic systems rarely operate continuously within such bounds. As detailed in the respective sections above, these assumptions take concrete and consequential forms in plant applications. In each case, they can be theoretically motivated but ultimately require experimental validation, which is often lacking or technically difficult to obtain. A key weakness of current plant-systems-engineering approaches is the limited observability of metabolic and redox state variables in vivo. This gap is being addressed by genetically encoded live-cell biosensors, such as roGFP2-based probes for redox and metabolite imaging, which enable spatiotemporal resolution of metabolic states in plant cells (Nietzel et al. 2019), or ratiometric Matryoshka-type biosensors, which enable quantitative, in vivo monitoring of metabolites and transporter activity (Ast et al. 2017).

This biological complexity necessitates strong assumptions when applying engineering tools. Classical beam theory assumes homogeneous materials under small deformations, conditions rarely met in fluttering canopies or plant stands, rendering predictions of leaf position for light interception approximations. Control theory is similarly constrained: classical approaches focus on linear time-invariant systems and single-input single-output systems, whereas plants are neither linear nor single-channel. Applying control engineering tools, therefore, frequently requires linearization and assumptions of zeroth- or first-order kinetics, which are theoretically defensible but require experimental validation. Some control tools also need accurate physical models of metabolic processes, which remain unavailable because of poorly characterized reaction mechanisms and missing parameters. Computational fluid dynamics faces its own limitations: the Navier–Stokes equations are not analytically solvable for complex flow scenarios. Simulations in plants rely on numerical simulations that depend on detailed meshes of vascular or cellular geometry. Mesh errors can produce incorrect results, and numerical solvers are computationally costly and may fail to converge. Stochasticity arising from genetics, development, and environmental context often deviates from the statistical assumptions underlying classical engineering tools. As a consequence, the direct transfer of engineering methodologies can require exploration of high-dimensional parameter spaces and careful reinterpretation of model assumptions.

Yet these limitations also reveal **opportunities** when engineering tools are applied selectively. Engineering approaches can help classify plant metabolic input–output relationships when mechanisms are incompletely known, identify dominant dynamic orders, infer potential feedback structures, and support modular reasoning about metabolic and transport networks. These outputs can help to build accurate models of plant metabolism despite knowledge gaps in hybrid modeling or digital twin frameworks. Thus, the application of engineering tools could lead to more accurate predictions of crop yields or land use in climate change scenarios. Analogies between engineered systems and systems in plants can stimulate hypothesis generation and guide experimental design, particularly when researchers work with unfamiliar organisms or novel environmental conditions. At a broader scale, these frameworks form the foundation for hybrid modeling strategies that embed data-driven components within mechanistic structure and for digital-twin approaches that could enable predictive modeling of crop performance under dynamic field conditions.

Realizing these opportunities requires awareness of genuine **threats** that accompany uncritical transfer. It remains uncertain to what extent methodologies developed for purpose-built technical systems are fully transferable to plants, organisms shaped by evolutionary rather than design processes. There is a genuine risk that biological systems may be forced into engineering-style frameworks primarily because they are mathematically convenient, rather than biologically appropriate. In addition, incomplete biological knowledge and limited measurement accessibility can lead to extensive parameter exploration, increased computational cost, and potentially misleading interpretations if data quality is insufficient. Engineering tools may appear rigorous and attractive even when the empirical foundation does not justify their application. Finally, ethical or legal boundaries may further constrain the translation of engineering interventions to living plants.

Navigating these trade-offs requires selective application rather than universal translation. Engineering approaches prove most valuable when system architecture matters more than precise parameters (controller motifs, network topology), when physical constraints dominate biological complexity (beam mechanics, fluid transport), and when dynamic behavior must be characterized systematically (frequency-domain analysis, transfer functions). Conversely, caution is warranted when biological nonlinearity or stochasticity fundamentally violates core assumptions, when parameter values are inaccessible, and models of plant metabolism become underconstrained, or when engineering optimization criteria (efficiency, minimality) conflict with evolutionary outcomes (robustness, redundancy). Among the opportunities identified, integrating mechanistic engineering frameworks with machine learning—through hybrid models and digital twins—represents the most tractable route to overcome the weaknesses identified above.

## Outlook

Open questions

- How do plants implement robust feedback control of metabolism under realistic, fluctuating environments?
- What are the dominant time scales and “bottleneck modes” in plant metabolic regulation, and can they be identified from limited measurements in a control-theoretic way?
- Can we infer internal metabolic states from limited measurements in a control-theoretic way?
- How do the mechanical properties of tissues constrain and shape metabolic performance over time?
- How does network architecture (venation, xylem/phloem topology) constrain the flexibility of metabolic allocation, considering the dynamics of the demand?
- What are the design trade-offs plants solve between robustness, efficiency, and flexibility, and do engineering optimality criteria predict them?
- How do we design experiments that are maximally informative for engineering-style models, given the measurement constraints?

Together, the engineering frameworks reviewed here point to a set of open questions in plant metabolism, summarized in the *Open Questions Box*. Addressing them requires experimental designs and analytical approaches that explicitly embrace dynamics, feedback, and physical constraints, areas in which engineering methodologies offer a particular quantitative toolbox. Critically, these frameworks indicate not only *which measurements* are most informative, but also *which experiments* to prioritize and *how much data* is sufficient. In plant biology, this logic is already visible in practical identifiability analyses of plant growth models (Velluet et al. 2024), Bayesian topology inference from Arabidopsis time-series data (Broz et al. 2024), and coupled leaf-metabolism models (Töpfer 2021).

To distinguish between alternative controller architectures, time-resolved perturbation experiments tracking both controlled variable and regulatory intermediates are needed; steady-state metabolomics alone cannot resolve this. To validate beam-theoretic predictions, material properties and deformation must be measured under defined loads simultaneously. To test transport models, spatially resolved measurements of flow velocities and concentration gradients are needed rather than bulk tissue averages.

Bayesian optimization offers a principled framework for this, deciding which experiment to run next to most efficiently resolve model parameters under realistic data budgets, an approach already demonstrated in bioprocess engineering (Siska et al. 2026) and directly transferable to plant systems. Optimal experimental design and Fisher information analysis identify which perturbations and measurements will most efficiently resolve parameters that matter for a given model structure (Raue et al. 2009). Bayesian identifiability analysis extends this further: it asks whether competing model structures can be distinguished given a realistic measurement budget (Beck and Yuen 2004). If they cannot, the answer is not to collect more data of the same kind but to redesign the experiment. Applied to plant metabolism, these approaches could sharpen how we study dynamic regulation: rather than broad metabolomics snapshots, we would ask which metabolite time courses, at which perturbation frequencies and amplitudes, carry the most information about feedback topology. The iterative design-test-refine cycle, central to engineering practice, provides a template for turning these formal criteria into experimental protocols.

Mechanistic models, improved physical measurements, and hybrid computational methods are therefore making it possible to understand how plants operate under fluctuating field conditions. Engineering concepts provide a route for integrating these advances because they make assumptions explicit, quantify uncertainty, and define the physical limits within which biological regulation occurs. As plant science increasingly adopts quantitative and computational approaches, the distinction between mechanistic and data-driven models is likely to blur, leading to hybrid frameworks that integrate physical insight with empirical flexibility. The most useful models will combine physical insight with empirical flexibility, allowing plant metabolism to be studied not as isolated pathways, but as a constrained, dynamic system operating across scales.

## Data Availability

This is a review and there are no resources to be provided.
